# Selection for early and late adult emergence alters the rate of pre-adult development in *Drosophila melanogaster*

**DOI:** 10.1186/1471-213X-6-57

**Published:** 2006-11-28

**Authors:** Shailesh Kumar, Koustubh M Vaze, Dhanya Kumar, Vijay K Sharma

**Affiliations:** 1 Chronobiology Laboratory, Evolutionary and Organismal Biology Unit, Jawaharlal Nehru Centre for Advanced Scientific Research, P. O. Box 6436, Jakkur, Bangalore 560064, Karnataka, India

## Abstract

**Background:**

Circadian clocks have been implicated in the regulation of pre-adult development of fruit flies *Drosophila melanogaster*. It is believed that faster clocks speed up development and slower clocks slow it down. We established three sets of *D. melanogaster *populations (*early*, *control *and *late*). The *early *and *late *populations were raised by selecting for flies that emerged either in the morning or in the evening under 12:12 hr light/dark (LD) cycles. After 75 generations of selection, the time course and waveform of the adult emergence and activity rhythms of the *early *and the *late *populations diverged from each other as well as from the *controls*. In this paper, we report the consequence of this selection on the rate of pre-adult development.

**Results:**

We assayed the pre-adult development time of the selected and control populations under 12:12 hr LD cycles and constant darkness (DD). Under LD cycles, the *early *populations develop faster than the *controls*, while the *late *populations develop slower than the *controls*. Although flies take longer to develop under DD than in LD, the relative differences between the mean development times of the selected and control populations remain unaltered in DD. In a separate experiment designed to investigate the effect of time of egg collection and experimental conditions on the duration of pre-adult stage, we assayed the development time of the selected and control populations by collecting eggs at different times of the day (morning and evening) and by assaying their pre-adult development time under constant light (LL), LD, and DD conditions. Irrespective of the time of egg collection and assay light regime, the *late *flies continue to develop slower than the *early *flies.

**Conclusion:**

The results of our study clearly indicate that selection on the timing of adult emergence alters the rate of pre-adult development in *D. melanogaster*. The timing of egg collection as well as assay light regime does not have any measurable effect on the relative differences between the developmental rates of the *early *and the *late *flies. Taken together these results appear to suggest that pleiotropic effects of clock genes mediate correlated changes in the timing of adult emergence and the rate of pre-adult development in *D. melanogaster*.

## Background

Circadian (*circa *= about; *dies *= day) clocks enable organisms to adapt to ambient environmental conditions by coupling behavioral and physiological events to cyclic factors in the environment [1,2]. Timing of such events functions towards maximizing organism's potential to survive under fluctuating environmental conditions, suggesting a role of circadian clocks in the regulation of life history traits [3-6]. Circadian clocks have been implicated in the regulation of pre-adult development time and adult life span in a few insect species including fruit flies *Drosophila melanogaster*. Faster clocks are believed to speed up development and shorten life span, while slower clocks are thought to slow down development and lengthen life span [5-7]. For example, in a study on the *period *(*per*) mutants of *Drosophila*, it was shown that the *per*^*S *^flies (with a free-running period (τ) of ~19 hr) develop faster than the wild type flies (τ~24 hr), while the wild type flies develop faster than the *per*^*L *^flies (τ~28 hr) [7]. Similarly, in a study on the melon flies *Bactrocera cucurbitae*, where faster and slower developing lines were created through laboratory selection, it was shown that the τ of eclosion rhythm of the faster developing line was shorter (~22.6 hr) than that of the slower developing line (~30.9 hr) [8-10].

In *Drosophila*, the timing of adult emergence depends upon the developmental state of the flies, the phase and the period of their developmental clocks, and upon the ambient environmental condition [11-13]. Consequently, certain times of the day form a "forbidden zone" for emergence, while a narrow window of time constitutes the "allowed zone" or "gate" of emergence [11-15]. It is believed that a continuously consulted circadian clock "reads" the developmental state of the flies, and only those that are mature enough to emerge during the gate are allowed to emerge, while others are made to wait until the next gate opens.

Under 12:12 hr LD cycles, the adult emergence in *Drosophila *like many other insects, follows a bimodal pattern; most flies emerge at dawn, while a small fraction of them emerge during the dusk with little or no emergence for the rest of the day [15-17]. Such patterns of emergence have been previously used to derive the "early" and "late" strains of *D. pseudoobscura *[14], *Pectinophora gossypiella *[18] and *D. melanogaster *[19]. These strains were derived by selecting for flies that emerged during the morning (lights-on) and the evening (lights-off) hours under 12:12 hr LD cycles. As a result, the peak of emergence in the early and the late strains diverge from the parental strains, and the selected strains show a correlated change in the τ of their emergence rhythm in DD. Although, the peak as well as the τ of the emergence rhythm diverged among the selected strains, their light-induced phase responses curve (PRC, a plot of phase shifts in a rhythm as a function of the phase of light pulse exposure) remain strikingly similar. The authors interpreted their results in the light of a "master-slave oscillator" model. They argued that the differences in the phase and the period of the emergence rhythm of the early and late strains were not due to altered circadian pacemakers, but due to altered coupling between the master (circadian pacemaker) and slave oscillators underlying eclosion rhythm. Although, it is possible to obtain phase separation of the emergence peaks through altered coupling between the constituent oscillators, it is hard to imagine how similar circadian pacemakers can generate oscillations with widely different period. Previous studies on the early and the late strains of *Drosophila *were mainly aimed at studying the response of selection on the circadian phenotypes, and therefore correlated changes in the life history traits were never examined. Given that the early and late populations differ from each other as well as the controls in terms of their circadian phenotypes, it would be interesting to investigate the consequence of selection on the timing of adult emergence on the rate of pre-adult development.

Part of the problem in drawing meaningful conclusion from previous selection studies on the early and late emergence is the lack of sufficient description of the starting population, population size, experimental condition and selection strategy. In addition, in most previous studies the unit of replication used was individual not population, which makes it difficult to rule out the possibility that the divergence in the emergence patterns in the selected strains was not due to inbreeding and/or random genetic drift. Individuals live, reproduce and die, and as a consequence of heritable differences in reproductive output among individuals, populations evolve. Therefore, the unit of replication in any study addressing evolutionary question should be population not individuals, as it is the genetic composition of a population that changes over time in an adaptive manner.

In this paper, we report the results of our experiments designed to study the effect of selection for the timing of adult emergence on the rate of pre-adult development in fruit flies *D. melanogaster*. Four population each of *early*, *control *and *late *were derived from four large, outbred, random mating *Drosophila *populations, which were reared under 12:12 hr LD cycles for several generations [20]. After 75 generations, the pre-adult development time of the selected and the control populations was assayed under LD and DD conditions. Further, in order to investigate the effect of timing of egg collection and experimental conditions on the rate of pre-adult development, the development time was assayed under constant bright light (LL), LD and DD conditions by collecting eggs from the selected and control flies during the morning (close to "lights-on") and the evening (close to "lights-off") hours. The results of these experiments are expected to provide an estimate of the intrinsic rate of pre-adult development in the presence and absence of circadian gating. For example, in DD, the pre-adult development time is known to be governed by circadian gate (associated with clocks) [21], while in LL condition circadian gating is completely abolished, and therefore time taken by the flies to complete pre-adult development in LL would solely depend upon the intrinsic rate of development. The results provide interesting insights into the link between circadian rhythms and pre-adult developmental time in fruit flies *D. melanogaster*.

## Results

### Experiment 1

**Figure 1:**
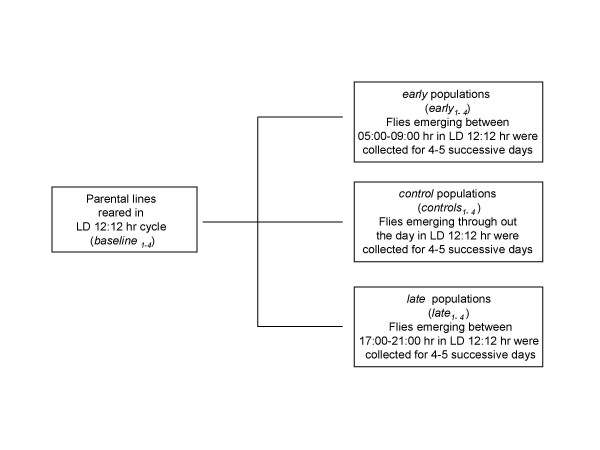
Schematic representation of the selection protocol. The selection was carried out under 12:12 hr LD cycles ("lights-on" at 08:00 and "lights-off" at 20:00 hr). Baseline populations (baseline_*1..4*_), maintained for several generations under LD cycles were used to derive four early (*early*_*1..4*_) and four late (*late*_*1..4*_) populations by imposing selection on the timing of adult emergence. Four control populations (*control*_*1..4*_) were also derived simultaneously from the baseline populations, which did not experience any conscious selection pressure. Flies emerging during the morning hours (05:00–09:00 hr) were used to create the *early *populations, those emerging during the evening hours (17:00–21:00 hr) were used to create the *late *populations, while flies emerging through out the day were used to raise the *controls*.

**Figure 2:**
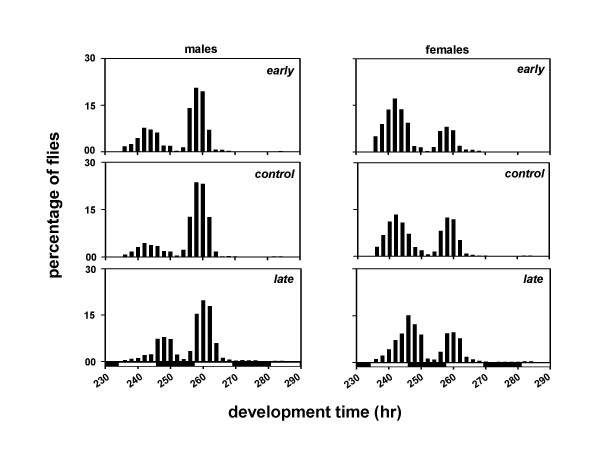
Eclosion profiles of the *early, control *and *late *populations under 12:12 hr LD cycles. Time in hours is plotted along the x-axis and percentage of flies is plotted along the y-axis. The eclosion profiles of males and females are shown in the left and right panels, respectively. Filled and empty bars indicate the light and dark phases of the LD cycle.

**Figure 3:**
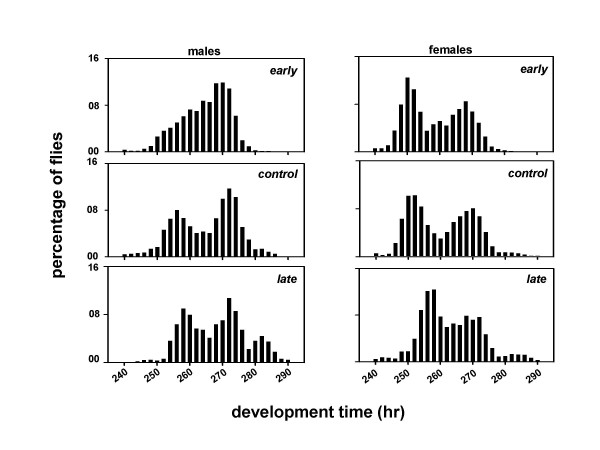
Eclosion profiles of the *early, control *and *late *populations under DD. Other details are same as in Figure 2.

We derived three sets of *Drosophila *populations by imposing selection for timing of adult emergence on four baseline populations which were maintained for several generations under 12:12 hr LD cycles. Each set consists of four matched pairs of populations derived from the parental baseline populations (Figure 1). After 75 generations of selection, the pre-adult development time of the males and females was estimated under LD and DD conditions. Females from all three sets of populations develop faster than the males, and flies take longer to develop under DD than LD (Table 1; Figures 2, 3). Under LD as well as DD, the *early *populations develop faster than the *controls*, while the *late *populations develop slower than the *controls *(Table 1; Figures 2, 3).

**Table 1:** Mean development time of the *early, control *and *late *populations

**Light regime**	**Population**	**Sex**	**Mean ± SEM**
LD	***early***	M	251.58 ± 0.65
		F	244.28 ± 0.65
	***control***	M	253.98 ± 0.49
		F	247.32 ± 0.76
	***late***	M	254.62 ± 0.64
		F	249.73 ± 0.91
			
DD	***early***	M	262.16 ± 0.83
		F	257.29 ± 1.13
	***control***	M	2.62.59 ± 1.15
		F	258.61 ± 1.22
	***late***	M	265.96 ± 0.98
		F	261.29 ± 0.95

Mean (± SEM) development time of males (M) and females (F) from the selected and control populations under light/dark (LD) and constant dark (DD) conditions. Pre-adult development time of a fly (in hours) was calculated as the time interval between the midpoint of egg collection window and the midpoint of the 2 hr period during which the fly emerged as adult.

A composite mixed model analysis of variance (ANOVA) on the mean development time data revealed a significant main effect of population (*F*_*2,6 *_= 27.65, *p *< 0.01), light regime (*F*_*1,3 *_= 11.01, *p *< 0.05), and sex (*F*_*1,3 *_= 579.26, *p *< 0.001) (Table 2). Post-hoc comparisons using 95% confidence interval (95%CI) around the mean revealed that the development time is significantly shorter under LD than DD, and the mean development time of the females is significantly shorter than the males (Tables 1, 2; Figures 2, 3). Post-hoc multiple comparisons using 95%CI revealed that the development time of the *early *populations is significantly shorter than the *controls*, while that of the *late *populations is significantly longer than the *controls *(Tables 1, 2; Figures 2, 3). Further, the effect of population × light regime, population × sex, light regime × sex, and population × light regime × sex interactions are not statistically significant (Table 2), which suggests that the relative differences between the development time of the males and females of the three populations remain comparable under LD and DD regimes.

**Table 2:** Analysis of variance (ANOVA) on the development time data

	***df *Effect**	**MS Effect**	***df *Error**	**MS Error**	***F***	***p*-level**
**Population (P)**	2	66.66	6	2.41	27.64	**0.001**
**Light regime (L)**	1	1469.41	3	133.52	11.01	**0.045**
**Sex (S)**	1	349.15	3	0.60	579.26	**0.001**
**Block (B)**	3	198.17	0	0	--	
**P × L**	2	3.85	6	1.29	2.98	0.126
**P × S**	2	1.72	6	1.95	0.88	0.462
**L × S**	1	9.52	3	3.53	2.69	0.199
**P × B**	6	2.41	0	0	--	--
**L × B**	3	133.51	0	0	--	--
**S × B**	3	0.60	0	0	--	--
**P × L × S**	2	1.82	6	0.771	2.37	0.175
**P × L × B**	6	1.29	0	0	--	--
**P × S × B**	6	1.95	0	0	--	--
**L × S × B**	3	3.53	0	0	--	--
**P × L × S × B**	6	0.77	0	0	--	--

Results of analysis of variance (ANOVA) on the development time data obtained from the assays done under light/dark (LD) and constant dark (DD) conditions. In the ANOVA design, population (P), light regime (L) and sex (S) were used as fixed factors, whereas replicate or Block (B) was used as random factor. In all cases the replicate means were used as the unit of analysis and hence, only the fixed factor could be tested for significance. Statistically significant values (*p *< 0.05) are shown in bold.

The profiles of the adult emergence indicate that the developmental rates of the selected populations have diverged from each other as well as from the controls (Figures 2, 3). Although, the differences between the development rates of the *early*, *control *and *late *populations appear to be consistent across both LD and DD, they did not reach statistical levels of significance in a *Kolmogorov-Smirnov *test for two samples.

### Experiment 2

To investigate the effect of phase of egg collection and assay light regimes on the pre-adult development time, we assayed the development time of the *early*, *control *and *late *populations under LL, LD, and DD. This was done under 12:12 hr LD cycles by collecting eggs from the selected as well as control populations in a 2 hr window during the morning and evening hours. The development time of the *early *populations is essentially indistinguishable from the *controls *under all light regimes except DD, nevertheless, the *late *populations clearly take several hours longer to develop than the *early *and *controls *(Table 3).

**Table 3:** Mean development time of the *early, control *and *late *populations

**Light regime**	**Population**	**Sex**	**Mean ± SEM**	
			
			**morning**	**evening**
LL	*early*	M	234.04 ± 0.90	239.18 ± 0.63
		F	230.42 ± 0.31	235.46 ± 0.54
	*control*	M	234.84 ± 0.81	239.82 ± 0.44
		F	230.90 ± 0.81	235.44 ± 0.50
	*late*	M	239.20 ± 1.00	246.26 ± 1.06
		F	236.09 ± 1.33	242.39 ± 0.68
LD	*early*	M	251.50 ± 1.49	255.76 ± 0.44
		F	246.66 ± 1.66	252.81 ± 0.66
	*control*	M	251.84 ± 1.04	255.38 ± 0.57
		F	246.22 ± 0.77	253.13 ± 1.11
	*late*	M	254.80 ± 0.81	259.30 ± 0.91
		F	250.55 ± 0.77	256.09 ± 0.79
DD	*early*	M	270.91 ± 0.86	268.46 ± 0.68
		F	267.25 ± 1.19	263.75 ± 0.85
	*control*	M	269.98 ± 0.56	270.31 ± 0.45
		F	263.76 ± 0.69	266.27 ± 0.45
	*late*	M	273.76 ± 0.73	274.97 ± 0.31
		F	269.29 ± 0.78	270.94 ± 0.61

Mean (± SEM) development time of males (M) and females (F) from the selected and control populations under constant light (LL), light/dark (LD) and constant dark (DD) conditions. The eggs were collected in a 2 hr window during the morning (07:00–09:00 hr) and the evening hours (17:00–19:00 hr). Pre-adult development time (in hours) was calculated as the time interval between the midpoint of egg collection windows and the midpoint of the 2 hr period during which the fly emerged as adult.

**Figure 4:**
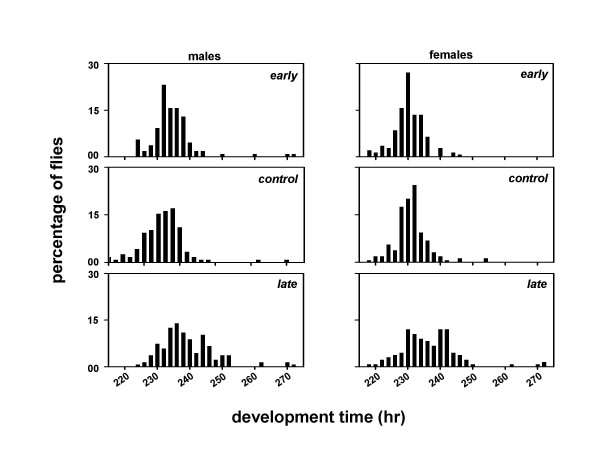
Eclosion profiles of the *early, control *and *late *populations under LL. Other details are same as in Figure 2, except that the eggs were collected in a 2 hr window during the morning hours (07:00–09:00 hr).

**Figure 5:**
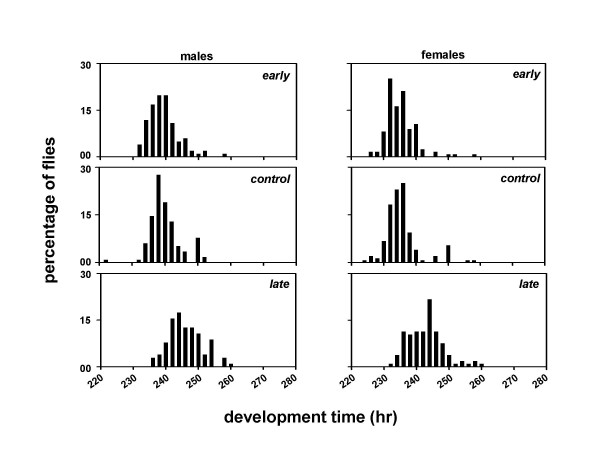
Eclosion profiles of the *early, control *and *late *populations under LL. Other details are same as in Figure 2, except that the eggs were collected in a 2 hr window during the evening hours (17:00–19:00 hr).

**Figure 6:**
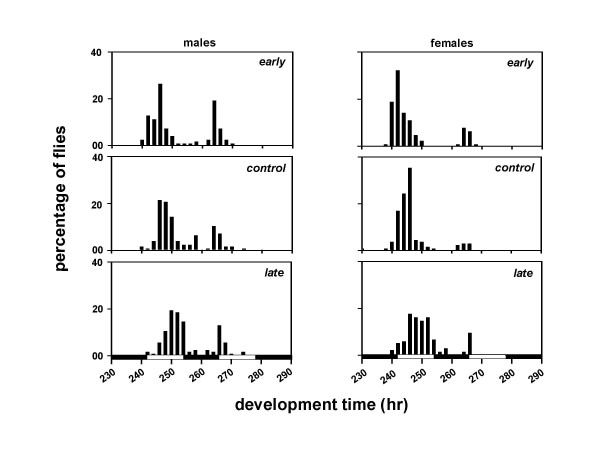
Eclosion profiles of the *early, control *and *late *populations under LD. Other details are same as in Figure 2, except that the eggs were collected in a 2 hr window during the morning hours (07:00–09:00 hr). Filled and empty bars indicate the light and dark phases of the LD cycle.

**Figure 7:**
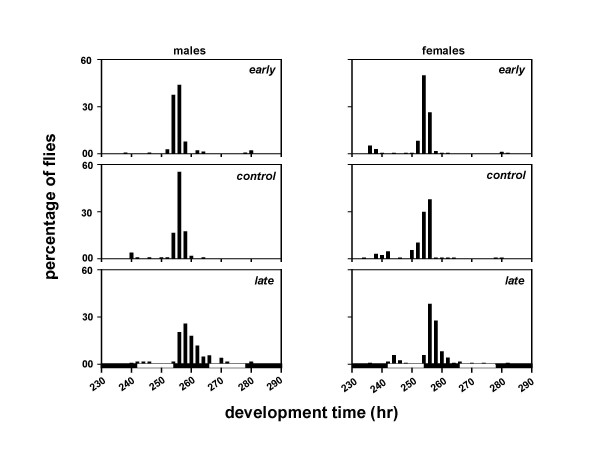
Eclosion profiles of the *early, control *and *late *populations under LD. Other details are same as in Figure 2, except that the eggs were collected in a 2 hr window during the evening hours (17:00–19:00 hr). Filled and empty bars indicate the light and dark phases of the LD cycle.

**Figure 8:**
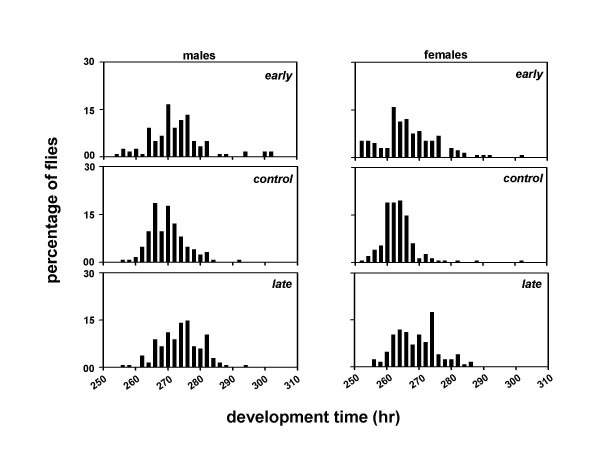
Eclosion profiles of the *early, control *and *late *populations under DD. Other details are same as in Figure 2, except that the eggs were collected in a 2 hr window during the morning hours (07:00–09:00 hr).

**Figure 9:**
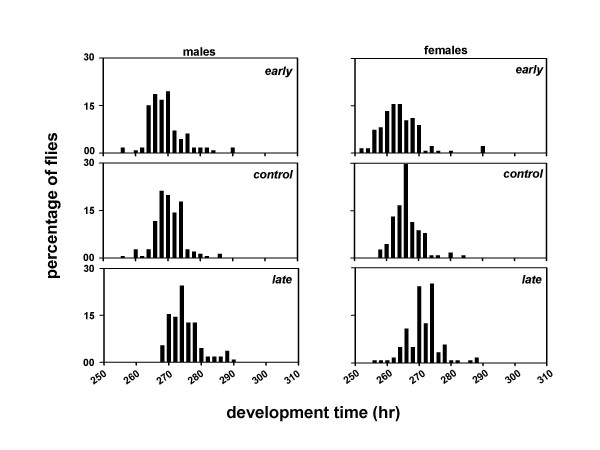
Eclosion profiles of the *early, control *and *late *populations under DD conditions. Other details are same as in Figure 2, except that the eggs were collected in a 2 hr window during the evening hours (17:00–19:00 hr).

A composite mixed model analysis of variance (ANOVA) on the mean development time data revealed a significant main effect of population (*F*_*2,306 *_= 120.85, *p *< 0.001), light regime (*F*_*2,306 *_= 4203.08, *p *< 0.001), sex (*F*_*1,306 *_= 200.37, *p *< 0.001), and egg collection window (*F*_*1,306 *_= 153.07, *p *< 0.001) (Tables 3, 4). Post-hoc multiple comparisons using Newman-Keuls test revealed that the development time is shortest under LL, followed by LD and DD, in that order (Figures 4, 5, 6, 7, 8, 9). The development time of females is significantly shorter than the males. Multiple comparisons also revealed that, irrespective of the time of egg collection and assay light conditions, the development time of the *late *flies is significantly longer than the *early *and *controls *(Figures 4, 5, 6, 7, 8, 9; Tables 3, 4). The development time of the *early *and *controls *is comparable under all light regimes except DD, where the *early *flies develop significantly faster than the *controls*.

**Table 4:** Analysis of variance (ANOVA) on the development time data

	***df *Effect**	**MS Effect**	***F***	***p*-level**
**Population (P)**	2	839.29	120.85	**0.001**
**Light regime (L)**	2	29189.57	4203.08	**0.001**
**Sex (S)**	1	1391.51	200.37	**0.001**
**Window (W)**	1	1063.04	153.07	**0.001**
**L × P**	4	19.58	2.82	**0.025**
**L × W**	2	275.76	39.71	**0.001**
**P × W**	2	27.83	4.01	**0.019**
**L × S**	2	4.86	0.70	0.497
**P × S**	2	2.78	0.40	0.670
**W × S**	1	11.25	1.62	0.204
**L × P × W**	4	21.83	3.14	**0.015**
**L × P × S**	4	0.54	0.08	0.989
**L × W × S**	2	11.44	1.65	0.194
**P × W × S**	2	5.03	0.72	0.486
**L × P × W × S**	4	2.33	0.34	0.854

Results of analysis of variance (ANOVA) on the development time data obtained from the assays done under constant light (LL), light/dark (LD) and constant dark (DD) conditions. In the ANOVA design, population (P), light regime (L), sex (S) and window of egg collection (W) were used as fixed factors. Statistically significant values (*p *< 0.05) are shown in bold.

## Discussion

In an earlier study on the same populations, we had shown that after 55 generations of selection on the timing of adult emergence the peak of emergence of the *early *and *late *populations diverged by about 4–5 hr, which suggests that *D. melanogaster *populations respond to selection by evolving different timing for their emergence behaviour (*Shailesh Kumar*, *Dhanya Kumar*, *Dhanashree Paranjpe*, *C R Akarsh and Vijay Kumar Sharma*, *unpublished manuscript*). In the present study, we show that the *early *populations develop significantly faster than the *controls*, while the *late *populations develop significantly slower than the *controls*, which suggests that the pre-adult development time of the selected populations is altered as a correlated response to selection on the timing of adult emergence. Consistent divergence in the rates of pre-adult development among four sets of replicate populations, which were treated through one generation of common rearing condition, clearly implies selection as the cause, as it is unlikely that all four replicate populations would simultaneously undergo similar sequence of genetic changes through random genetic drift.

Under LD cycles, the difference between the mean development time of the *early *and *late *populations closely matches the phase separation between the peaks of their emergence rhythm (Figures 2, 6, 7), thus suggesting that the "favourable phase" of emergence coincides with the "appropriate development state" of the flies to produce gated emergence. These results are in good agreement with the findings of an earlier study by Qiu and Hardin [12]. In this study, the short period mutants (*per*^*S*^) of *Drosophila *were found to develop faster than the wild type flies, because the *per*^*S *^mutants encountered a favorable gate of emergence much earlier than the wild type flies [12]. This is also consistent with the fact that the emergence peak of the *per*^*S *^flies precedes lights-on, whereas that of wild type flies follows lights-on [22]. These studies thus suggest that, the development time of *Drosophila *depends upon the developmental state of the flies, their developmental clocks, and the timing of lights-on/lights-off in a LD cycle [12].

In a separate set of experiments when we assayed the development time of the flies under LL, LD and DD conditions by collecting eggs at two different times of the day, the values of the development time of the *early*, *control *and *late *flies changed marginally. However, irrespective of the time of egg collection and assay light conditions, the *late *flies continue to develop slower than the *early *and *controls*. Although, in this experiment the development time of the *early *populations is essentially indistinguishable from the unselected *controls*, under all regimes except DD – a different result from the *experiment 1*, in which the *early *populations develop significantly faster than the *controls *in both LD and DD, the *late *populations clearly take several hours longer to develop than the *early *and *controls *– consistent with a developmental delay rather than merely an effect of circadian gating. These results thus circumvent any influence of phase of the LD cycle, and/or circadian gating on the rate of pre-adult development in the *early*, *control *and *late *flies. It is particularly interesting to note that the relative differences between the developmental rates of the *early *and *late *flies are maintained even under bright LL, where circadian gating is clearly abolished [see additional file 1]. This completely rules out the possibility that the observed differences in the rates of pre-adult development among the *early*, *control *and *late *populations are due to circadian gating. Taken together the results of our experiments suggest that the *early *and *late *populations have evolved different rates of pre-adult development as a consequence of selection on the timing of adult emergence, and that similar genetic changes may underlie the timing of adult emergence and pre-adult development time in *D. melanogaster*.

Given the fact that circadian clocks have no measurable effect on the rate of pre-adult development in the *early*, *control *and *late *populations, our study further suggests that the connection between circadian clocks and development time may not be causal, but could be mediated through pleiotropic effects of clock genes on circadian rhythm and pre-adult development time. Pleiotropic effects are not entirely uncommon in *Drosophila *circadian literature, as they were reported in an earlier study on the *per *mutants [7]. In this study, the development time and the circadian period were found to be positively correlated; the *per*^*S *^flies developed faster than the wild type flies, and the *per*^*L *^flies developed slower than their wild type counterparts. Changing environmental conditions (DD, very bright light (VLL), LD 12:12 hr and LD 12:12 hr with superimposed temperature cycles) did not alter the nature of the correlation, and short and long period flies continued to develop faster and slower than the wild type flies. Pleiotropic effects of the clock genes were also implicated in previous studies that involved selection for faster and slower pre-adult development in the melon fly *Bactrocera cucurbitae *[8-10]. In these studies, the development time was found to be positively correlated with the time of mating and circadian period. The circadian period of faster developing line was shorter (τ~22.6 hr) than the slower developing line (τ~30.9 hr), and mating in the faster developing line occurred earlier than the slower developing line [8-10].

In a separate study, designed to bypass the pleiotropic effects of clock genes, the pre-adult development time of four populations of *Drosophila *was assayed under different LD cycles in conjunction with the adult emergence rhythm [23]. In this study, the eclosion rhythm was speeded up or slowed down using short (20 hr) or long (28 hr) LD cycles [23]. As a consequence, the development time of the flies was either shortened or lengthened compared to those observed under 24 hr LD cycles, suggesting that periodicity of the LD cycles, and/or circadian rhythm regulates the rate of pre-adult development in *Drosophila*.

## Conclusion

The results of our study clearly demonstrate that selection for early and late emergence alters the timing of emergence peak, and causes an associated change in the rate of pre-adult development, suggesting a genetic correlation between the timing of adult emergence and pre-adult development time in *D. melanogaster*. Although, distinct genetic changes causing parallel changes in the timing of adult emergence and the rate of development, as a result of imposed selection can not be ruled out without a determination of the genetic changes leading to both phenotypes, it is likely that similar genetic changes underlie both phenotypes. Nonetheless, it is also possible that complex and less understood interactions of a number of factors such as available gate of emergence, circadian period, assessment of developmental state, ambient light intensity, temperature, and humidly, regulate key life history traits such as pre-adult development time in *Drosophila*.

## Methods

### Fly stock population generation and maintenance

The *early *and *late *populations were initiated from four ancestral baseline populations of *D. melanogaster*. The maintenance protocol and ancestry of the baseline populations are described in details in Sheeba et al [20]. Briefly, they were maintained as independent evolutionary entities for several generations under alternating 12:12 hr LD cycles (light intensity 15 ± 5 μW/cm^2^/sec), prior to the initiation of the selection experiment. Temperature (25 ± 1°C) and humidity (75 ± 5%) were maintained constant throughout the experiment, and banana-jaggery food and water was available *ad libitum*. A total of ~1200 breeding adults per population, with roughly equal number of males and females, were maintained in Plexiglass cages (25 cm × 20 cm × 15 cm) with abundant food, on a 21 day discrete generation cycle. Eggs were collected on the 21^st ^day after the previous egg collection by placing petri dishes with food into these cages for 2 hr (between 09:00–11:00 hr), and then dispensed into glass vials (18 cm height × 2.4 cm diameter) at a density of about 300 eggs per vial with 10 ml of food. Between the 9^th ^to 13^th ^days after egg collection, freshly emerged flies were collected into Plexiglass cages containing a petri dish of food. On the 18^th ^day, a generous smear of yeast-acetic acid paste was applied on the food plates and kept in the cages. Three days later, eggs were collected from these flies to initiate the next generation. From the four baseline populations, four populations of early (*early*_*1..4*_), and four populations of late (*late*_*1..4*_) flies were initiated by imposing selection for early and late adult emergence. Four control populations (*control*_*1..4*_) were also initiated simultaneously, which did not experience any conscious selection pressure (Figure 1). Each *early*, *control *and *late *population was derived from one baseline population, thus forming matched selected and control pair (*early*_*i*_, *control*_*i *_and *late*_*i *_are more closely related than *early*_*j*_, *control*_*j *_and *late*_*j*_, *i*,*j *= 1–4). For starting the selected populations, eggs of approximately same age were collected from the four baseline populations (*baseline*_*1–4*_) on the 21^st ^day after the previous egg collection by placing petri dishes with food into the cages for 2 hr (between 09:00–11:00 hr) (Figure 1). The eggs were then dispensed at a density of about 300 eggs into vials (18 cm height × 2.4 cm diameter) with 10 ml of food. Nine days later, freshly emerged flies were collected for 4–5 successive days into Plexiglass cages containing a petri dish of food. Flies for the *early *and *late *populations were collected between 05:00–09:00 hr and 17:00–21:00 hr, respectively, while those for the *controls *were collected throughout the day. This selection scheme continued for 75 generations under 12:12 hr LD cycles, where lights came on at 08:00 hr and went off at 20:00 hr (Figure 1). Flies emerging for 4–5 successive days were particularly selected to rule out any possibility of inadvertent selection for faster and slower development.

### Standardization of the selected populations

Imposition of different maintenance regimes may induce non-genetic parental effects. Therefore, all selected and control populations were subjected to one generation of common rearing condition prior to the developmental time assays, during which no conscious selection pressure was imposed. Eggs for all three populations (*early*, *control *and *late*) were collected from the running cultures by placing petri dishes with food into cages for 2 hr (between 09:00–11:00 hr) and dispensed into vials at a density of about 300 eggs per vial (18 cm height × 2.4 cm diameter) with about 10 ml of food. On the 12^th ^day after egg collection, all flies were collected into Plexiglass cages with abundant food. The progeny of these flies hereafter will be referred as the standardized flies.

### Pre-adult development time assays

#### Experiment 1

After 75 generations of selection, the pre-adult development time of the selected and control populations was assayed. From each of the standardized replicate populations (*early*_*1..4*_, *control*_*1..4 *_and *late*_*1..4*_) eggs laid on banana medium over a 2 hr window (between 09:00–11:00 hr) under LD cycles (lights-on at 08:00 hr and lights-off at 20:00 hr) were collected. Exactly 30 eggs were dispensed into long vials containing ~6 ml banana food and 20 such vials were set up from each population. Ten vials from each replicate population were introduced into DD and the remaining ten vials into LD. Thus a total of 240 vials were used for the assays (10 vials × 4 replicates × 2 light regimes × 3 populations). Fluorescent white light of 15 ± 5 μW/cm^2^/sec intensity was used during the light phase of LD cycles and red light of λ > 650 nm was used during DD as well as the dark phase of LD cycles. Temperature and relative humidity under LD and DD regimes were monitored continuously using Quartz Precision Thermo-Hygrograph, Isuzu Seisakusho Co, LTD and were found to be constant. The vials were regularly monitored for emergence once the pupae became dark. Emerging adults were collected every 2 hr, sexed and counted. This continued until no flies emerged for 3 consecutive days. The mean pre-adult development time was estimated for each vial. The development time of a fly in hours was calculated as the time interval between the midpoint of egg collection window and the midpoint of 2 hr period during which the fly emerged as adult.

#### Experiment 2

In a separate set of experiments, the pre-adult development time of the selected and control populations was assayed under LL, LD and DD by collecting eggs over a period of 2 hr in the morning (between 07:00–09:00 hr) and evening (between 17:00–19:00 hr) from one of the standardized replicate populations (*early*_*1*_, *control*_*1 *_and *late*_*1*_) kept under 12:12 hr LD cycles (lights-on at 08:00 hr and lights-off at 20:00 hr). Fluorescent light of 15 ± 5 μW/cm^2^/sec intensity was used during LL and the light portion of LD cycles, whereas red light of λ > 650 nm was used during DD and the dark portion of LD cycles. The light intensity during LL was maintained bright enough to abolish circadian rhythmicity of adult emergence [see additional file 1]. Other details are described in *Experiment 1*.

### Statistical analyses

Data from the development time assays were subjected to composite mixed model analysis of variance (ANOVA). For *experiment 1*, replicate populations (or blocks) were treated as random factor, whereas population, light regime, and sex were fixed factors crossed with replicate. In all cases the replicate means were used as units of analysis and hence, only the fixed factor could be tested for significance. Post-hoc multiple comparisons were done using 95% confidence interval (95%CI) around the mean. For *experiment 2*, population, light regime, sex and egg collection window were treated as fixed factors. Post-hoc multiple comparisons were done using Newman-Keuls test. The emergence waveforms of the selected populations were analyzed using *Kolmogorov-Smirnov test*. All analyses were implemented using Statistica for Windows [24].

## Authors' contributions

SK, KMV and DK performed the experiments described and analyzed the data. VKS conceived the study and provided valuable suggestions throughout the study. All authors read and approved the manuscript.

## Supplementary Material

Additional file 1 Eclosion assay under constant light (LL) condition. The data provided demonstrates that circadian rhythm of adult emergence is abolished in fruit flies *D. melanogaster *in LL. Click here for file
