# Supplementary material for: Selection for early and late adult emergence alters the rate of pre-adult development in Drosophila melanogaster
Source: BMC Dev Biol. 2006 Nov 28;6:57. doi: 10.1186/1471-213X-6-57 (PMC1693556; doi:10.1186/1471-213X-6-57)
Supplement: Additional file 1 — Eclosion assay under constant light (LL) condition. The data provided demonstrates that circadian rhythm of adult emergence is abolished in fruit flies D. melanogaster in LL. [file 1471-213X-6-57-S1.doc]

**Additional File S1**

***Eclosion assay under constant light (LL) condition***

The waveform of adult emergence under constant light conditions (LL, with light intensity 15 ± 5 μW/cm^2^/sec.) was assessed for flies from the *control* populations to assess the state of circadian clocks under bright LL. For this assay, eggs laid on banana medium over a 2 hr window (09:00-11:00 hr) in 12:12 hr LD cycles (lights-on at 08:00 hr and lights-off at 20:00 hr) were collected from the standardized flies and dispensed at approximately at 300 eggs per vial into vials with 10 ml of food, and kept under LL. Ten such vials were set up. These vials were monitored for the first emergence and thereafter checked regularly at every 2 hr interval for eight consecutive days and the number of adult flies was recorded. The time series data thus obtained was subjected to Fourier spectral analysis using Statistica*^TM^* [1]. Statistical significance of contributions from periodicities in the periodogram was tested using Siegel’s modification of the Fischer test [2]. This method delineated the periods present in the time series data by defining a threshold value at *p* < 0.05 [3].

**Figure S1** Eclosion waveform of flies from the control populations assayed under LL (constant light) of light intensity of about 15 ± 5 μW/cm^2^/sec. Number of flies emerging at every 2 hr interval is plotted along the y-axis and time since first emergence is plotted in hours along the x-axis. The corresponding periodogram reveals that adult emergence is arrhythmic in LL. Periodicity in hour is shown along the x-axis and fractional contribution of each periodicity is shown along the y-axis.

# References

1. StatSoft Inc. STATISTICA^TM^ Vol I **General conventions and statistics**. Tulsa; *StatSoft* Inc. 1995.
2. Siegel FJ: **Testing for periodicity in a time series.** *Am Stat Assoc* 1980, **75**:345-348.
3. Rao AK, Sharma VK: **A** **simple approach for the computation of multiple periodicities in biological time series.** *Biol Rhythm Res* 2002, **33:**487-502.
